# Reusable SERS Platform of Femtosecond Laser Processed Substrate for Detection of Malachite Green

**DOI:** 10.3390/molecules29215053

**Published:** 2024-10-26

**Authors:** Yinghao Lun, Bing Zhao, Yuanhai Geng, Wenhan Du, Xiaona Zhao, Xuan Wang

**Affiliations:** School of Remote Sensing and Information Engineering, Wuhan University, Wuhan 430072, China; lunyinghaoedu@163.com (Y.L.); zhaobing@whu.edu.cn (B.Z.); yuanhaigeng@whu.edu.cn (Y.G.); wenhan.du@whu.edu.cn (W.D.)

**Keywords:** SERS substrates, femtosecond laser, microstructured silicon, long-term stability, recyclability, reusability performance

## Abstract

This study presents the development of highly efficient Surface-Enhanced Raman Scattering (SERS) substrates through femtosecond (fs) laser processing of crystalline silicon (Si), resulting in mountain-like microstructures. These microstructures, when decorated with gold nanoparticles (Au NPs), exhibit remarkable SERS performance due to the creation of concentrated hotspots. The enhanced Raman signals originate from the excitation of localized surface plasmon resonance (LSPR) of the Au NPs and the multi-scale rough morphology of the Si substrates. Finite-element method simulations confirm the electromagnetic field enhancement in narrow gaps, supporting the experimental observations. The fabricated substrates show high uniformity, oxidation resistance, long-term stability, and exceptional reproducibility, making them ideal for molecular detection, especially in food safety applications. A remarkable enhancement factor (EF) of 10^10^ is attained in the detection of Malachite Green (MG), boasting a limit of detection (LoD) as low as 10^−14^ M. This underscores the immense potential of this technique for achieving highly sensitive and dependable SERS-based sensing capabilities.

## 1. Introduction

As society continues to evolve and advance, the significance of food safety has become increasingly paramount. Ensuring the safety and quality of our food supply chain is not just a matter of health and well-being, but also a cornerstone of sustainable development and societal progress. Malachite Green (MG, C_23_H_25_CN_2_), a prominent triarylmethane compound, was once widely employed as an effective bactericide in the aquaculture industry [1,2]. However, extensive research has revealed its detrimental effects, including teratogenicity [3,4], carcinogenicity, and mutagenicity towards human beings [5,6,7], prompting numerous nations to ban its use in aquaculture and it is not approved by the US Food and Drug Administration [8,9]. Despite these stringent regulations, MG continues to be illicitly employed within the industry owing to its low cost and exceptional efficacy, posing a significant challenge to regulatory authorities and public health, so a rapid and stable method for detecting microorganisms is urgent [10].

Usually, the prevailing methods for detecting MG in aquatic products heavily rely on techniques such as liquid chromatography [11,12], liquid chromatography-mass spectrometry, and gas chromatography-mass spectrometry. However, these methodologies are often plagued by drawbacks like time-consuming procedures and intricate operational requirements [13,14,15]. Consequently, there is a pressing need to develop a rapid, specific, and accurate method for detecting MG, addressing the limitations of current techniques. Surface-enhanced Raman scattering (SERS) is a highly sensitive and nondestructive method for detecting molecules via the enormous enhanced Raman signal intensity of molecules adsorbed on rough metal surface or on colloidal particles. To cater to the escalating market demands, numerous nanofabrication techniques have been established to fabricate SERS substrates [16]. Specifically, tailored for real-world applications, the core challenges for these SERS substrates lie in achieving exceptional performance across fabrication efficiency, cost-effectiveness, sensitivity, uniformity, stability, and versatility [17]. The most commonly employed methods, ion beam irradiation and electron beam lithography, though effective, are hindered by several drawbacks. For instance, these techniques are associated with high costs, involve multistage procedures, offer low throughput, and are frequently time-consuming, limiting their scalability and efficiency [18,19]. In comparison to the aforementioned lithographic techniques, femtosecond (fs) laser ablation, with its unique one-step, maskless, direct, and non-contact approach, has garnered considerable attention as a promising technology for fabricating functional micro-devices, notably SERS substrates. It offers a highly efficient and alternative method for designing intricate micro- and nano-structures over extensive areas on a wide range of solid substrates [20]. Crucially, fs laser ablation distinguishes itself by producing high-quality surface structures with exceptional accuracy due to minimized heat-affected zones, and it achieves a lower ablation threshold under identical processing conditions compared to long pulse ablation [21].

Herein, we propose a mountain-like microstructure formed on a silicon (Si) surface through fs laser processing as a SERS platform, and further demonstrate that these mountain-like microstructures, when combined with subsequently deposited gold nanoparticles (Au NPs), can embody the required randomness and roughness to accommodate a high hotspot density. This surface roughness enables more efficient coupling of surface plasmons with far-field light, thereby generating stronger and more uniform electromagnetic (EM) fields. The mountain-like microstructured Si can be converted into SERS substrates with high enhanced factors (EFs), and most importantly, serve as a reusable platform for molecular detection since they can maintain moderately active even after 20 repeated cycles. The study highlights the potential of fs laser-processed microstructured Si substrates combined with Au NPs as a highly efficient and reusable SERS platform for environmental monitoring and other analytical applications. The unique properties of the substrates, including their structural stability, long-term durability, and recyclability, make them promising candidates for widespread adoption in various fields.

## 2. Results and Discussion

Here, we delve into the characteristics of the mountain-like microstructures processed onto Si through fs laser processing, emphasizing the surface morphological features and the enhanced SERS response achieved subsequent to decoration with Au NPs. Notably, the focus of our SERS experiments revolves primarily around detecting toxic or hazardous molecules in food products. Specifically, we have chosen the highly representative probe molecules of MG, an antiparasitic medication that bioaccumulates in seafood, to demonstrate the effectiveness of our approach. As SERS platforms, the substrates exhibit remarkable performance, showcasing their potential as powerful tools for food safety analysis.

### 2.1. Structure and Morphological Characteristics

**Figure 1:**
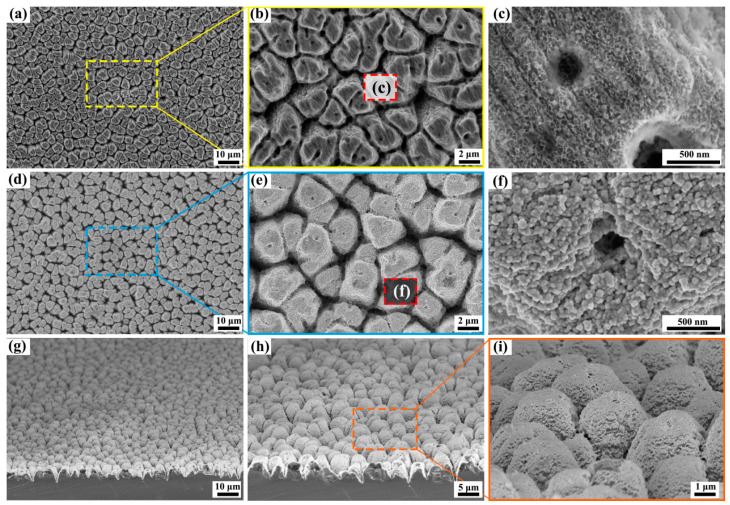
SEM micrographs of the fs laser ablated microstructures on the Si surface at different magnified views (**a**–**c**) generated by using the laser fluence *F*: 10.7 mJ/cm^2^ under water; SEM images of the microstructures after depositing 80 nm Au NPs (**d**–**f**); panels (**g**–**i**) report a 70° rotation view of the corresponding SEM images of the microstructures after depositing Au NPs.

The Si-based samples, designed specifically for enhancing SERS response, underwent rigorous processing and were subsequently characterized for their morphological features. This was accomplished through detailed SEM micrographs and 3D microscope images, providing a comprehensive understanding of their structural properties. Figure 1a–c present the SEM images of Si substrates that have undergone fs laser irradiation prior to the deposition of Au NPs. As evident from the images, the processed Si surface exhibits irregular blocky structures characterized by long-range order amidst short-range disorder, with the orientation randomly scattered across the surface. Each block, with a diameter ranging approximately from 2 to 3 μm, is distinctly separated by steep valleys. The surface of these blocks exhibits extreme roughness, giving the impression of being adorned with a layer of intricate flocculent microstructures. Upon depositing a uniform Au NPs (80 nm) as reported in Figure 1d–f, the entire surface of the processed Si becomes exceptionally bright. Moreover, as evident from the magnified view presented in panel (f), the surface is now densely populated with Au NPs, with the size ranging from several to up to 100 nm can be found, which have agglomerated in a fluffy, clustered manner. According to previous reports, the EM field enhancement of the closely spaced interacting metal particles is about thousands of times of the isolated single nanosphere [22,23]. Therefore, with the processed rough Si surface serving as a supportive base, the Au NPs deposited upon it exhibit significantly enlarged specific surface area, which produces an abundant of closely packed nanoparticles.

Furthermore, the presence of numerous narrow gaps among these Au NPs facilitates the excitation of the local surface plasmon resonance (LSPR) effect, ultimately leading to the formation of hotspots. This optimized configuration enhances the overall performance and functionality of the material [24]. It has been proven that these hotspots, which form in gaps between or within metal nanostructures, can yield enhanced Raman properties.

Figure 1g–i report the 70° rotation view of the corresponding SEM images of the substrates after Au NPs depositing, that clearly illustrate the depths of the micro-patterns, herein the block body displayed in the top view has now become the shape of a “Mongolian yurt” in the side view. Each microstructure has an arc-shaped top with roughly the same height, and the surface is covered with a layer of shiny Au NPs. It has been well-documented that the seamless interface between Si semiconductor and Au metal fosters a robust interaction between the semiconductor’s electronic states and the dielectric-confined EM modes inherent in the Au metal [24]. This synergy triggers a dynamic charge transfer process from the metal to the semiconductor, proficiently inhibiting the undesirable recombination of electron-hole pairs. As a result, the exciton-plasmon coupling phenomenon meticulously localizes the electric field within the interface gap between these two materials, leading to a pronounced enhancement of the EM field, which is of paramount importance for various applications [23].

**Figure 2:**
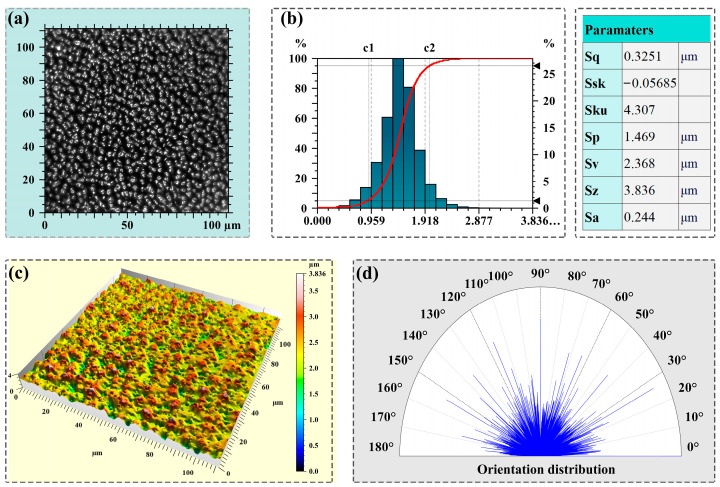
Confocal micrographs in 2D views of the microstructures processed by fs laser on Si (**a**), and the depth distribution histogram (**b**) and the calculated roughness parameters. 3D confocal micrographs images of the microstructures (**c**) and the corresponding orientation distribution of the microstructures on the Si surface (**d**).

Figure 2 further reports the morphological characterization via the 3D confocal microscope and comes with the surface roughness analysis. Panel (a) clearly showcases a two-dimensional (2D) confocal micrograph of the microstructures crafted on the Si using an fs laser. As observable, the processed Si surface exhibits a distinct pattern of tiny regions with alternating light and dark distributions, displaying an overall regular and uniform appearance. The luminous sections correspond to the raised structures, akin to the blocky areas previously mentioned, whereas the darker areas signify the slits situated at the junctures of these blocky regions. This darker signal distribution in the slits is attributed to their relatively distant positioning from the confocal microscope’s lens, coupled with inadequate light penetration into these narrow crevices. Furthermore, the statistical histogram of the relative height (depth) of the processed Si surface is shown in panel (b). As depicted, the relative height exhibits a normal distribution that is symmetric about an axis at 1.5 μm, with a symmetrical distribution on both sides of the axis. The processed Si substrates are multi-branched with many microstructures as well as narrow gaps on the surface, and thus possessing a wide range of light scattering and absorption properties [25], which are favorable for obtaining a higher SERS signal due to the integration of electric charge in large curvatures of the microstructures [26].

Moreover, the roughness parameters, which have been computed from the 3D confocal microscope images, are depicted on the right-hand side of panel (b). Here, the roughness parameters, *Sa* and *Sq*, are employed to assess surface roughness, with *Sa* representing the average height deviation and *Sq* revealing the root mean square height. Given the prevalence of micro/nano structures and particles with diverse sizes and shapes, the adoption of statistical metrics akin to mean surface grain radius, such as root mean square roughness, becomes imperative. Specifically, *Sku* quantifies the sharpness of the roughness shape, and a value of *Sku* = 4.3 > 3 signifies a prominently mountainous structural profile. Furthermore, *Ssk* determines the tendency of the roughness shape, classifying it as concave, symmetric, or convex. Since *Ssk* = −0.0569 < 0, this indicates that after laser processing, the height distribution lies predominantly above the average surface level, pointing towards a peak-like structural configuration.

Panel (c) presents the 3D confocal micrographs of the Si-based sample. As anticipated, the substrate surface distinctly displays mountain-like structures, imparting a relatively rough texture to the entire plane. Panel (d) reveals the orientation distribution of the microstructures on the processed Si, exhibiting an approximate isotropic orientation. Notably, the directional coherence of these microstructures is significantly disrupted due to the re-deposited nanoparticulate debris arising from fs laser ablation in air. This phenomenon plays a pivotal role in the formation of above-wavelength quasi-periodic grooves, ultimately influencing the orientation distribution [27].

### 2.2. SERS Evaluation and LoDs Calculation

SERS has emerged as a powerful analytical technique due to its ability to provide vibrational fingerprints of molecules with high sensitivity and specificity. This technique leverages the enhanced Raman scattering phenomenon that occurs when molecules are adsorbed on certain metal surfaces, such as gold or silver. In recent years, there has been a growing interest in developing SERS substrates for the detection of MG, given the need for rapid, on-site analysis in forensic and law enforcement applications. The integration of nanotechnology, particularly in the form of metal nanoparticles, has significantly improved the performance of SERS substrates in terms of sensitivity, reproducibility, and stability.

In this study, we aim to evaluate the performance of Si SERS substrates for the detection of ten MG. By analyzing the limit of detection (LoDs) and dynamic range, aiming to identify the most promising substrates for further development and optimization. Additionally, the importance of reproducibility and stability, as these factors are crucial for the practical application of SERS in MG detection, has been discussed later.

As mentioned above, the enhancement of Raman signal is commonly attributed to the proliferation of hotspots, which arise from the Localized Surface Plasmon Resonance (LSPR) excited by densely packed Au NPs. In this context, the mountain-like substrates, intricately adorned with Au NPs on their surfaces, act as reservoirs for a copious amount of these hotspots. These abundant hotspots serve to dramatically amplify the EM fields, ultimately leading to a notable enhancement in signal intensity. Therefore, the surface properties of the SERS substrate were also investigated in detail in terms of stability, reusability and homogeneity. Consequently, when probe molecules are drawn into these hotspots, they experience a dramatic enhancement of the EM field, ultimately enhancing the Raman signal performance.

**Figure 3:**
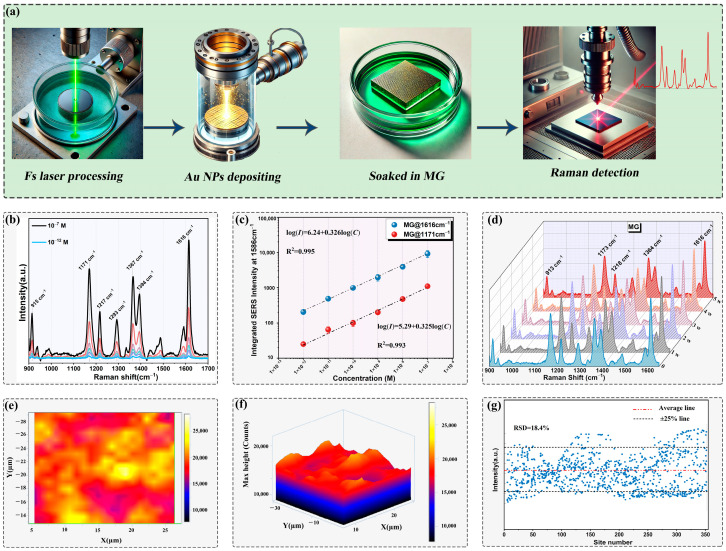
Diagram of the preparation and characterization of the microstructured Si substrates and the Raman detection (**a**); Raman spectra of Au coated microstructured Si substrates on MG (**b**) at different concentrations for the determination of corresponding LoDs; the corresponding relationship between the intensity of the 1171 cm^−1^ and 1616 cm^−1^ SERS peaks of adenosine and the concentration of molecules solution (**c**), at 638 nm Raman excitation wavelengths. Raman spectra for 10^−7^ M of MG randomly collected periodically during a period of 5-week exposure in air (**d**). SERS signal maps (1616 cm^−1^ peak) for 10^−7^ M of MG solutions collected from microstructured Si substrate in panel view (**e**) and 3D view (**f**), the scanning step is 1 µm in the X and Y directions; and the corresponding distribution of the Raman intensities from all the 1000 spots (**g**).

Figure 3a illustrates the comprehensive process for the preparation and characterization of microstructured Si substrates, along with the implementation of Raman detection. As illustrated, the Si substrates underwent initial processing with an fs laser. Following this, a layer of Au NPs with the thickness of 80 nm was deposited onto the processed Si surface through ion sputtering. Afterwards, the Si substrates modified with Au NPs were submerged in the MG solution for a duration of 12 h. Lastly, SERS detection was conducted utilizing Raman spectroscopy, with a focus on the soft palate.

The Raman spectra for MG solutions with different concentrations (ranging from 10^−7^ M to 10^−12^ M) under the illumination of a 638 nm laser beam on the mountain-like microstructures have been acquired and depicted in Figure 3b. As can be distinguished, the band attributed to the N-C and C-C bond stretching at 1616 cm^−1^, the scattering band attributed to the ring C-H stretching at 1171 cm^−1^ and 1293 cm^−1^, the band attributed to the N-C stretching at 1367 cm^−1^ and the bands attributed to the C-C/C-H vibration at 1394 cm^−1^ were obtained. Moreover, the band attributed to the ring skeletal radial vibration was obtained at 915 cm^−1^ [27,28]. The strongest characteristic peak of MG is at 1616 cm^−1^, indicating the different vibrational modes of its phenyl ring [29]. And since the original 1619 cm^−1^ peak overlapped with the 1594 cm^−1^ peak, thus causing a left shift of the peak position to 1616 cm^−1^ [29]. As illustrated, the intensity of the 1619 cm^−1^ peak declined continuously as the concentration went down. And the mean peak intensity decreased to an intensity value slightly above the testing noise, and according to the method by [30], we concluded the LoDs was 10^−14^ M (MG) using this substrate.

In SERS analysis, the calibration curve serves as a bridge connecting the analytical signal (such as Raman intensity) to the concentration of the analyte. However, traditional linear calibration curves often fail to accurately describe the concentration-signal relationship in complex systems, especially within the low concentration range. This is because the SERS effect itself is influenced by various factors, such as surface roughness, hot spot distribution, and molecular interactions with the surface, all of which can lead to the emergence of nonlinear relationships [30]. To accurately assess the LoDs in SERS analysis, it is crucial to deeply understand and appropriately handle this nonlinear relationship. This paper aims to introduce a novel SERS substrate—a microstructure Si substrate processed by fs laser combined with Au NPs, and to discuss in detail the characteristics of its nonlinear calibration curve, providing a more precise and reliable methodological foundation for SERS analysis.

During the experiments, a pronounced nonlinear calibration curve was observed, which reflects the complex relationship between the SERS effect and the concentration of the analyte. The nonlinear calibration curve accounts for the complex relationship between the signal and concentration, which may arise from various factors such as saturation effects of the SERS substrate and the interaction mechanisms between the analyte and the substrate. In these scenarios, a simple linear model fails to accurately describe the relationship between the signal and concentration, necessitating the use of more complex nonlinear models for fitting [30]. According to the previous report, Langmuir adsorption isotherm model was employed the to describe the nonlinear relationship between the signal and concentration. The Langmuir model is a classic adsorption theory that assumes adsorption occurs on a monolayer surface and that there is no interaction between adsorption sites. This model can well describe many adsorption processes, including analyte adsorption in SERS. By utilizing nonlinear regression methods, the authors fitted the experimental data to the Langmuir model, obtaining a nonlinear calibration curve that describes the relationship between the signal and concentration. This curve not only enhances the accuracy of calibration but also provides a more reliable basis for subsequent LoDs assessment.

Additionally, under the optimized conditions, the linearity (an approximate linear range within a certain concentration range) for detection of MG was evaluated with the concentration of calibration standards against the SERS peak areas. Thus, an assessment of the Raman intensity versus concentration was conducted to ascertain the LoDs as well, with the results presented in Figure 3c. For comparative purposes, the integrated SERS intensities at 1616 cm^−1^ and 1171 cm^−1^, specific to N-C and C-C bond, and the ring C-H stretching of MG, have also been quantified and included in the analysis. As illustrated, the relationship between the probe molecules solution concentration and Raman intensities in logarithmic value is accurately depicted by well-fitted linear functions and plotted against *log*[*MG*]. As the analyte concentration increased, a proportional augmentation in SERS intensity was observed, allowing for the determination of the LoDs of the fabricated substrate to be as low as 10^−14^ M. In addition, the characteristic vibrational peaks situated at 1171 cm^−1^, 1367 cm^−1^, and 1616 cm^−1^ remained clearly distinguishable within the spectra, even at the significantly diminished concentration of 10^−12^ M, demonstrating the exceptional sensitivity and specificity of the SERS sensing platform for the detection of the target MG molecules. The peak intensity *I* increases exponentially with *log*(*C*), which could be perfectly fitted by a linear equation of *log*(*I*) = 6.24 + 0.326 *log*(*C*) with a correlation coefficient (*R*^2^) of 0.995 for the peak at 1616 cm^−1^, and *log*(*I*) = 5.29 + 0.325 *log*(*C*) with a correlation coefficient (*R*^2^) of 0.993 for the peak at 1171 cm^−1^ were obtained, respectively. The statistical analysis conducted on the experimental results, with a confidence level of 95%, suggests that no significant discrepancies exist between the proposed methodology and the reference method [27]. As consequence, the proposed method boasts a broader linear range and heightened sensitivity compared to other techniques, attributed to the high selectivity of the imprinted particles and the exceptional sensitivity of SERS. Notably, the cubic root dependence observed in our calibration curve is consistent with previous reports in the field of SERS [31,32,33]. For the linear range on the sigmoidal curve depicted in Figure 3c, a linear equation has been derived. It can be concluded that detection of MG can be carried out easily and selectively with high affinity and sensitivity using the laser processed SERS substrates.

Moreover, Figure 3d reports the Raman spectra registered for MG (10^−7^ M) on the rough substrates collected over a period of 5-week exposure in ambient air. As evidenced by the Raman spectra, even after a period of one month, the substrate retains a robust and uniform SERS intensity, closely mirroring their initial state, with notably no discernible decrease in the intensity of the Raman signals. This demonstrates the remarkable oxidation resistance and long-term stability of the fabricated SERS substrates. The enhanced and highly confined EM here based on the plasmon-enhanced Raman effect arisen from LSPR excited by the Au NPs on the rough substrates can achieve a potential application for the SERS. As an ideal SERS substrate for detecting specific chemical molecules, it is essential to possess excellent structural stability, enabling it to maintain the enhanced Raman signal for a prolonged period when exposed to the ambient environment.

Furthermore, the experiments on SERS mapping detection were conducted on the mountain-like substrate, across expansive areas of approximately 20 × 20 μm^2^ in order to investigate the uniformity. The spatial map of the SERS signals obtained from 10^−7^ M of MG is presented in Figure 3e. The mapping signal was obtained by integrating the signal intensity after baseline correction of the raw Raman mapping data over a width of ±5 cm^−1^. It can be depicted from the color scale that the substrate exhibits remarkably uniform SERS distribution across the whole surface, with the color here indicating the intensity of the Raman signal. The Raman mapping in 3D view showing in panel (f) can better reveal the profiles of relative variation of the Raman signal intensity among the detected area. Secondly, a relative standard deviation (RSD) value of 18.4% was yielded after 1000 randomly recorded spectra were analyzed for the processed substrates, which indicates the reliable signal homogeneity of the mountain-like microstructures, as shown in panel (g). With the Raman signal intensity within ±25% of the average intensity reaching to 95% of the overall selected samples.

### 2.3. Stability and Sensitivity of Repeated SERS Detection

Apart from possessing high EFs and spatially uniform, an ideal SERS substrate necessitates superior chemical stability, precise performance, rapid fabrication capabilities over extensive areas, and ultimately, exceptional reproducibility [34]. Therefore, to thoroughly evaluate the ubiquitous applicability of this SERS-based substrate, in addition to long-term stability assessments performed above, the cyclic reuse experiments were rigorously conducted as well.

**Figure 4:**
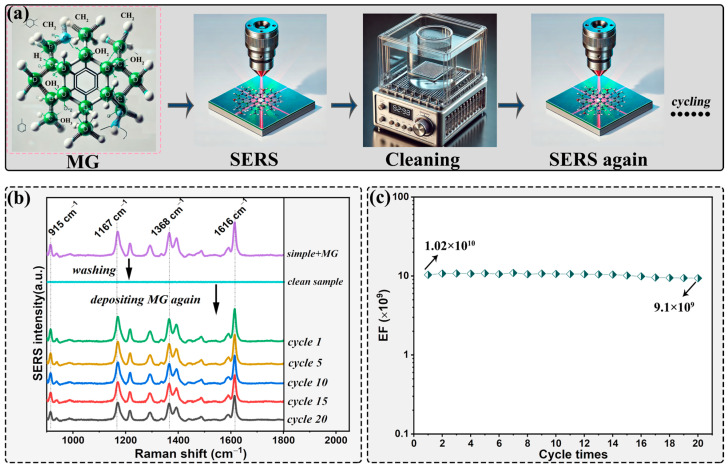
Diagram of the preparation and characterization of the microstructured Si substrates and the cycled Raman detection experiments (**a**); Raman spectra of microstructured Si on 10^−7^ M MG recycling for several times (**b**) and the corresponding EFs values for 20 cycles under 638 nm Raman excitation wavelength (**c**).

In these experiments, Raman detection was carried out for each test up to 20 cycles, after each cycle, the substrate is thoroughly cleaned using an ultrasonic cleaning, as depicted in Figure 4a. Specifically, the Raman spectra obtained from cycles 1st, 5th, 10th, and 20th were meticulously chosen for comparative analysis, as depicted in panel (c). After each cycle, it is evident that the probe molecules on the substrate are thoroughly removed, resulting in a spectrum that closely resembles a straight line devoid of any discernible signal. Subsequently, the recycling experiments were reiterated in this manner for a cumulative total of 20 cycles. Notably, throughout the cycle tests, there was virtually no discernible difference in the Raman spectra, and even after the completion of 20 cycles, the substrates continued to exhibit exceptional SERS performance, with each characteristic peak Raman being distinctly observable and identifiable. Panel (c) clearly depicts the calculated EFs values for the uniquely processed mountain-like substrates, presented through an illustrative dotted line diagram. Remarkably, these substrates exhibit an unparalleled stability in their regenerative SERS performance, maintaining up to 91% of their initial EFs value of 1.02 × 10^10^ even after undergoing 20 consecutive cycles, restoring the substrate to its original state for subsequent use, demonstrating a good reutilization ability of the Si-based substrates. Specifically, the EFs value of the substrate remains robust at 9.1 × 10^9^, a testament to its outstanding mechanical durability. The consistent intensity underscores the negligible degradation of the prepared SERS substrates across numerous reuse cycles, rendering them an exceptional choice for reliable and durable SERS applications. And this unique attribute positions the proposed SERS platform as an ideal contender for the detection of specific chemical molecules.

### 2.4. Simulation and Theoretical Background

**Figure 5:**
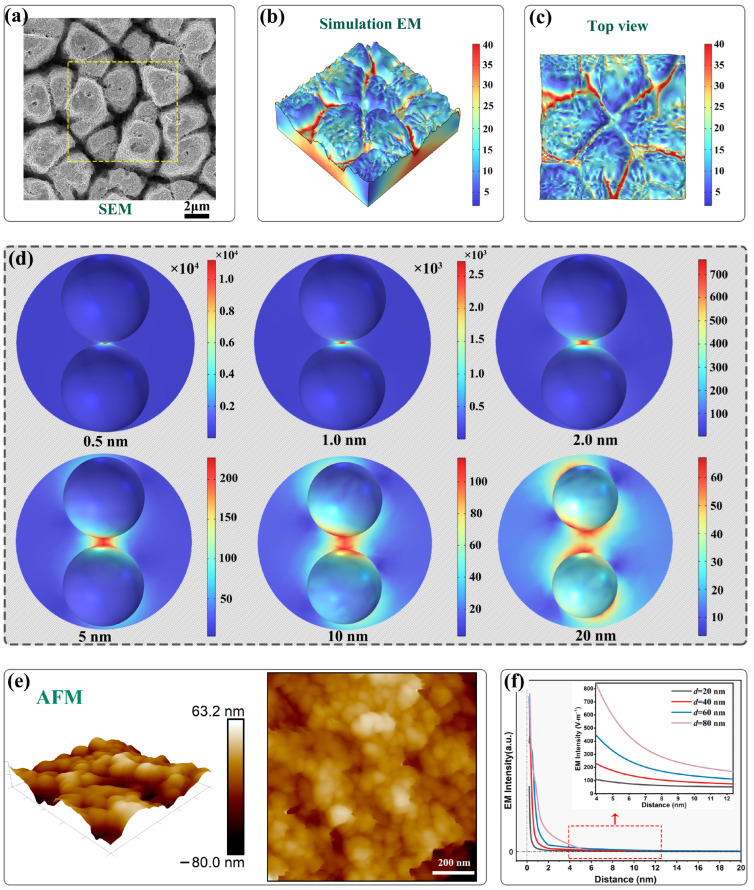
(**a**) SEM image depicting the mountain-like microstructures, serving as the zone guidance. (**b**) 3D simulation outcomes illustrating the intricate EM distributions across the model surface structures; (**c**) a top-down view of the simulated EM distributions; (**d**) enhanced exploration of EM interactions between two Au NPs, with the distance between them systematically varies from 0.5 nm to 20 nm; (**e**) AFM image showcasing the densely packed arrangement of deposited Au NPs on the microstructured substrate; (**f**) enhanced EM field profiles within the gaps between Au NPs of different particle size.

To unravel the underlying mechanism behind the enhanced EM intensity and gain a profound comprehension of the Raman response on the aforementioned mountain-like substrates, the finite element method (FEM) simulations was employed using the Maxwell solver in COMSOL Multiphysics (version 6.1). These simulations, which focused on model surface structures mirroring the morphological characteristics of the microstructures, were performed under a laser excitation wavelength of 638 nm. As depicted in Figure 5, the surface topography, acquired through SEM images combined with height information from the 3D microscope results above, served as the basis for the simulations. Arbitrary regions of 10 × 10 µm^2^ on the SEM images to model the surface morphological features for the FEM simulations, as shown in panel (a). Moreover, panels (b)~(c) present the 3D visualization of the EM intensity, adhering to false-color logarithmic scales legend displayed to the right of the images, with the maximum value of the color logarithmic scale set at 40 to clearly identify areas of the most pronounced enhancement. As evident from the visualization, the highly localized regions of intense EM field enhancement predominantly occur at narrow gaps, particularly between adjacent “yurts” (referring to the mountain-like structures). By correlating these findings with the Raman detection above, we can infer that the SERS mechanism is directly influenced by the tightening of the surface structures, with the narrow gaps acting as significant enhanced EM field region.

As reported [24] that the overall enhancement includes contributions from EM enhancement, mainly due to the plasmon excitation of metal nanostructures, and chemical enhancement, which originates from chemical interactions and photon-induced charge transfer between the metal and target molecule, and the former plays the major role. As for the deposited Au NPs, the resonance frequency can be tuned to the visible spectral regime by varying the configuration of the nanostructures. In this resonant state, free electrons transiently accumulate on the nanostructure surface and greatly increase the density of surface charges, leading to a tightly confined and strongly enhanced electric field on the surface [24]. The maximum excitation enhancement is achieved when the excitation wavelength exactly matches the LSPR peak position, here in our study, *λ* = 638 nm is the wavelength of the scattered light.

The deposited Au NPs on the surface further increase the roughness and thus providing with a multi-scale synergy effect due to the LSPR effect and the strong EM coupling. As reported that when two Au NPs approach each other to within a distance of 1~5 nm, a strong EM coupling may appear [35], which then greatly enhances the electric field intensity within the narrow gap, the regions with the strongest enhancement in the gaps are thus called hotspots. As depicted in Figure 5d, the electric-field enhancement within the hotspot decreases exponentially as the distance between the Au NPs increases and as one moves further away from the hotspot’s core along the nanoparticle surface, which results in a long-tail distribution surrounding the hotspot. It has been reported that for a 30-nm Ag nanoparticle dimer with a 2 nm gap, the surface area where the EFs exceed 1/10 of the maximum value comprises only 0.68% of the total surface area of the two spheres. Furthermore, just 0.59% of the total surface area accounts for 80% of the total signal intensity [36,37]. As evidenced by the AFM images in panel (e), maximizing the number of Au NPs that adhere fully to the substrate can promote the generation of additional hotspots. These densely packed Au NPs on the microstructures enhance attachment and, consequently, result in more efficient surface areas for SERS applications. Therefore, probe molecules trapped within the hotspot region can produce ultrahigh Raman signals compared to their free state. Additionally, when the analyte molecules are adsorbed onto the nanoparticle surface, their Raman scattering signals are amplified due to the intense localized optical field. Thus, the uniform electric field enhancement in the Au-deposited Si substrates ensures a high sensitivity of SERS detection in low concentration solution [29].

Furthermore, the enhanced EM field profiles within the gaps between Au NPs with the varying particle size were calculated and fitted, as shown in Figure 5f, emphasizing the role of gold atom particle size in modulating these fields. Corresponding to the display in panel (d), the EM intensity decreases exponentially as the distance between Au NPs increases, resulting in a long-tail distribution centered around the hot spot. As the diameter of the Au NPs increases from 20 nm to 80 nm, the EM intensity within the hot spots also intensifies, with a higher rate of increase observed for narrower inter-particle distances.

Therefore, it can be concluded that, on one hand, the rough surface of the substrates offers an abundance of active sites to AuNPs, facilitating their attachment and subsequently preventing aggregation. Additionally, the supported Au NPs composite materials provide a localized high concentration of Au NPs, allow greater ease of manipulation and recovery, and improved resistance to aggregation, which can extend the application to more harsh environments, including biological media [38]; one the other hand, the arrangement and organization of Au NPs on the mountain-like microstructured Si surface can allow the NPs optical properties to be tuned through surface plasmon coupling phenomena, and thus producing much more narrow gaps for achieving strong EM and hot spots [39]. As calculated by the COMSOL Multiphysics, the relative maximum EM fields of the rough substrates reaches as high as ≈5.68 × 10^5^, demonstrating that such a multi-scale rough morphology can largely contribute to the observed Raman enhancement, which likely ensues the plasmonic couplings induced by the ridges inherent to the mountain-like pattern. Since the Raman EFs are approximately proportional to the fourth power of the local EM field strength [40], the simulated EFs achieve a remarkable value of up to 5.68 × 10^9^, demonstrating a significant congruency with the experimental outcomes, which stand at 1.02 × 10^10^ and above. Moreover, the simulation results well illustrate the high density of the hot spots present at the narrow gaps, where the EM field increases considerably addressing a great effect of the LSPR. In summary, efficient SERS measurements require metal nanostructures with very narrow gaps, hot spots that are accessible to the target molecules and trapping of the target molecules in the hot spot [24].

## 3. Experimental Section

**Figure 6:**
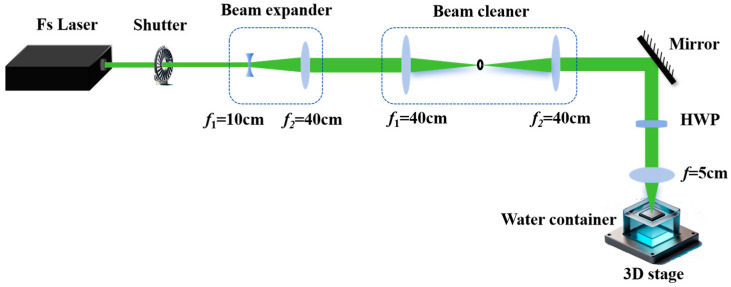
Schematic illustration of the fs laser underwater processing setup for the fabrication of microstructures on Si surface.

The experimental setup utilized for fabricating the SERS substrates via the fs laser processing technique is shown in Figure 6. Specifically, commercial N-type Si (111) wafers, double-side polished, with a thickness of 500 μm and resistivity ranging from 15–50 Ω·cm, were used as the targets. The target was affixed onto a 3D translation stage (MMC-100, Micronix, Fountain Valley, CA, USA), which boasts a minimum step size of 100 nm and a maximum translation speed of 10 mm/s. The laser pulses, with a duration of 200 fs and a wavelength of approximately 1030 nm, were generated by a Yb:KGW system (PHAROS-2mJ, Light Conversion, Vilnius, Lithuania). A frequency converter (HIRO PH1F3, Light Conversion, Vilnius, Lithuania) is employed to generate laser wavelength at 515 nm. By reducing the wavelength of the laser, a higher energy density at the focal point can be achieved, which is crucial for creating the desired textured microstructures on the Si surface. Additionally, the wavelength of 515 nm is more likely to be absorbed by the Si material than the wavelength of 1030 nm, leading to more efficient energy transfer and reduced thermal damage. The Si is processed under water environment by the fs laser, and the device is designed and fabricated by our group, and the corresponding schematic and details are reported in the previous work [41]. Moreover, by irradiating a stationary target with 100 laser pulses, we determined the energy threshold (*Eth*) to be 0.16 mJ (measured before the focus lens) and the fluence threshold (*Fth*) to be approximately 10.7 J·cm^−2^ (*Fth = Eth/(*$\pi \times {\omega_{0}}^{2}$)), respectively [42].

The morphology and depth of the processed microstructures were comprehensively analyzed using various microscopy techniques. Specifically, a Field Emission Scanning Electron Microscope (SEM, model JSM-7500F, JEOL, Tokyo, Japan), a three-dimensional (3D) confocal microscope (Smartproof 5, Carl Zeiss, Oberkochen, Germany), and an Atomic Force Microscope (AFM; SPM-9700HT, Shimadzu, Kyoto, Japan) were employed. Subsequently, a magnetron sputtering instrument (SD-900M, VPI, Beijing, China) was utilized to deposit Au NPs onto the sample surface. The equivalent Au layer thickness was precisely determined based on the sputtering deposition time. To characterize the SERS response of the laser-induced structures, a confocal Raman microscope (XploRA Plus, HORIBA Jobin Yvon, Oberursel, Germany) was employed. Raman measurements were conducted using a 50×/0.90 NA objective at near-infrared laser excitation wavelength of 638 nm. The excitation power was carefully adjusted to 40 mW, resulting in a power of around 4 mW at the sample surface. During the calculation of EFs, a specific volume of the solutions was dispensed onto the sample surface, and the covered area was accurately measured by 3D microscope. This approach allowed for the straightforward calculation of EFs values according to Luo’s method [24]. Malachite Green (MG, C_23_H_25_CN_2_) was obtained from Fuchen Chemical Reagent Factory (Tianjin, China).

To gain further insights into the EM field response and distribution on the elaborate SERS substrates, COMSOL Multiphysics version 6.1 was utilized for simulations. The geometry of the structures was directly reproduced from SEM and AFM images of the mountain-like microstructured Si surface, providing valuable insights into their EM properties.

## 4. Conclusions

This comprehensive study presents a highly effective and reusable SERS platform, leveraging fs laser-scribed ripple-like microstructures on crystalline Si surfaces combined with Au NPs. The resulting SERS substrate demonstrates exceptional reproducibility, sensitivity, and stability, with a remarkable enhancement factor of up to 10^10^ for MG detection, achieving a limit of detection as low as 10^−14^ M. The unique mountain-like microstructures, enriched with roughness and randomness, effectively accommodate numerous hotspots, amplifying the Raman signal intensity. Furthermore, the substrates exhibit robust reusability over multiple cycles with negligible degradation, retaining over 90% of its initial EFs after 20 cycles of use, highlighting their potential for practical applications in food safety and beyond. This work underscores the power of fs laser processing in creating sophisticated nanostructures for SERS, paving the way for the development of more sensitive and reliable molecular detection methods.

## Data Availability

Data are contained within the article.
